# *In vitro* micrografting success and histological union characterization of *Argania spinosa* (L.) skeels scion lines grafted onto rootstock G54

**DOI:** 10.3389/fpls.2026.1902844

**Published:** 2026-09-09

**Authors:** Hachimi Fathellah, Meryem Tahtah, Oudija Fatiha, Ilham Belkoura, Abdelaaty A. Shahat, Joe Miantezila Basilua, Omar M. Noman, Ibrahim Toufik

**Affiliations:** 1 Department of Biology, Laboratory of Innovative Materials and Biotechnologies of Natural Resources, Faculty of Sciences, Moulay Ismail University, Meknes, Morocco; 2 Department of Fundamental Sciences, Laboratory of In Vitro Culture, National School of Agriculture of Meknes, Meknes, Morocco; 3 Department of Biology, Laboratory of Ecology and Environment, Faculty of Sciences Ben M’sik, University Hassan II of Casablanca, Casablanca, Morocco; 4 Department of Pharmacognosy, College of Pharmacy, King Saud University, Riyadh, Saudi Arabia; 5 Department of Biostatistics and Mathematics, Faculty of Pharmacy, Paris Cité University, Paris, France

**Keywords:** *Argania spinosa*, genotype effect, graft compatibility, graft-union formation, micrografting, necrosis, scion–rootstock interaction, vascular differentiation

## Abstract

The argan tree (*Argania spinosa* L.) is ecologically and economically important but its propagation is difficult on a large scale due to difficulty in rooting and grafting. This study provides a preliminary protocol for rootstock preparation and micrografting, and evaluated the compatibility of three scion genotypes (G41, G27, G27/41) grafted onto the rootstock G54. *In vitro* seed germination on basal MS medium without plant growth regulators resulted in an 82% germination rate, resulting in a sufficient quantity of healthy rootstocks suitable for micrografting. Shoot regeneration trials identified substantial genotypic differences; G41 had the highest level of shoot regeneration (100%), at 0.1mg L⁻¹ GA_3_, and had the highest levels of vegetative vigor and branching potential, as confirmed by principal component analysis (PCA), whereas G27 displayed more limited morphogenetic performance. Micrografting compatibility differed significantly among the graft combinations. The G41/G54 combination achieved the highest graft success rate (80%) and the lowest necrosis rate (20%) (p < 0.001). Histological examination of G41/G54 at two months after grafting revealed callus continuity, cambial organization and lignified vascular elements spanning the graft interface, features consistent with anatomical graft-union formation. Under the tested conditions, grafting performance differed among the three scion lines grafted onto G54. Because only one rootstock genotype and one histologically examined graft combination were evaluated, these findings require validation through additional and reciprocal graft combinations, comparative histology, acclimatization and long-term survival assessment. The findings provide a preliminary basis for further development of argan micrografting protocols.

## 1 Introduction

*Argania spinosa* (L.) Skeels, or the argan tree, is endemic to North Africa (Msanda et al., 2005), predominantly distributed within the southwestern of Morocco, where it constitutes a significant component of agro-sylvo-pastoral systems in arid and semi-arid areas (Chakhchar et al., 2022; Santoro et al., 2023; Timzioura et al., 2025). The genus *Argania* belongs to the family Sapotaceae, and it has acquired several adaptations that have made it able to survive in environments with unpredictable rainfall patterns, high evapotranspiration rates (Amghar et al., 2021a; Amghar et al., 2021b; Ibrahim et al., 2026), and poor soil fertility, partly due to its extensive root system and ability to withstand drought stress (Oumasst et al., 2024). At present, approximately 830,000 hectares of argan forests are estimated to cover the species’ natural range, providing important ecosystem services, including soil stabilization (Aitlhaj, 2020). The argan tree has considerable socioeconomic value through the production of argan oil for food, cosmetics, and pharmaceutical applications, as well as the provision of fuelwood and livestock feed (Metougui et al., 2017).

However, A. spinosa populations have been increasingly affected by anthropogenic pressures, particularly overgrazing and overharvesting, which have contributed to reduced natural regeneration (Yamani et al., 2024; Moukrim et al., 2019). Conventional propagation by seeds is also challenging because of the species’ allogamous breeding system, which results in genetically heterogeneous progeny, long juvenility, and variable germination depending on seed biometric characteristics, storage duration, and pretreatment conditions (Mezghenni et al., 2014; Al-Menaie et al., 2007; Nouaim et al., 2002; Abdelaziz et al., 2014; Tahtah et al., 2026a; Toufik et al., 2026). These limitations highlight the need for efficient clonal propagation methods to facilitate the multiplication and conservation of selected elite genotypes.

*In vitro* propagation provides another avenue for the propagation of argan elites in a controlled environment. The techniques that could be used for propagation of argan include micrografting technique which involves combining the rooting ability of the rootstock and the genetics of a scion and has been proven useful in other woody recalcitrant species (Koufan et al., 2018). Nevertheless, the success of micrografting largely depends on the establishment of a functional graft union, which involves complex cellular and anatomical processes that remain insufficiently characterized in *Argania spinosa* (Lamaoui et al., 2019). Therefore, further investigation of grafting performance and graft-union formation is needed to improve the efficiency and reliability of micrografting protocols in this species.

Koufan et al. 2020a and Koufan et al. 2020b have proved the feasibility of micrografting *in vitro* of *Argania spinosa*, whereas the present study extends previous argan micrografting work by comparing three scion lines grafted onto a common G54 rootstock under identical experimental conditions. Unlike the previous study, which assessed the feasibility of micrografting using a single graft combination, the present work compares several scion genotypes, allowing genotype-dependent differences in graft success, necrosis, and morphogenetic performance to be identified. Furthermore, histological observations are integrated with grafting performance data to relate anatomical development at the graft union to grafting success. This combined approach provides further insight into the biological factors associated with graft compatibility and offers a scientific basis for evaluating scion–rootstock combinations for the clonal propagation of elite *Argania spinosa* germplasm. The best-performing combination, based on observed graft success, was further examined histologically to characterize graft-union formation.

In the present study, G54 was selected as the rootstock because seeds from this source were available in sufficient numbers and had shown suitable *in vitro* seedling development in preliminary culture observations, ensuring the availability of adequate and reproducible rootstock material for the micrografting experiments. In addition, the scion lines G41, G27, and G27/41 were selected based on their previous selection and maintenance *in vitro*, as well as their differing morphogenetic properties and propagation abilities, allowing the investigation of genotype-specific differences in grafting performance. G27/41 is a graft-derived scion line obtained from plantlets maintained *in vitro* following a previous micrografting experiment in which G27 and G41 were used as the scion and rootstock, respectively. The microcuttings used in the present study were excised from the *in vitro* shoots derived from the original G41 scion, above the previous graft-union region. Therefore, the G27/41 line is referred to here as a graft-derived scion line rather than a combined or genetically composite genotype. No genetic analysis was performed in the present study to determine whether the line retained the original G27 scion genotype or exhibited any graft-induced chimerism. This study aimed to produce *in vitro* G54 seedling rootstocks, compare the micrografting success of scion lines G41, G27, and G27/41 on G54 rootstock, and characterize the histological features of graft-union formation in the most compatible combination.

## 2 Materials and methods

### 2.1 Plant material

Mature fruits of Argania spinosa (L.) Skeels, collected from the G54 maternal tree/accession in a wild argan population in the Souss-Massa region of Morocco (Agadir, Morocco), were used to produce seedlings for use as rootstocks. The selected fruit were mature, uniformly mature, and void of any measurable external blemishes. The grafts examined in this study were produced from *in vitro*-grown microcuttings that had been maintained in the laboratory for four years in a controlled environment. These scions belonged to different genotypes: G27, G41, and the combined G27/41 (**Table 1**). The micrografting experiment was conducted at the National School of Agriculture in Meknes, Morocco.

**Table 1:** Summary of the rootstock–scion combinations, plant material origins, and morphological characteristics of the scions used in the *in vitro* micrografting experiments.

Graft combination	Rootstock genotype	Scion genotype	Origin of rootstock	Origin of scion	Scion length (mm)	Scion stem diameter (mm)
G41/G54	G54	G41	*In vitro* seedling	*In vitro* shoot derived from microcuttings maintained through successive subcultures	5–10	2–3
G27/G54	G54	G27	*In vitro* seedling	*In vitro* shoot derived from microcuttings maintained through successive subcultures	5–10	2–3
G27/41/G54	G54	G27/41	*In vitro* seedling	Graft-derived *in vitro* scion line maintained through successive subcultures	5–10	2–3

### 2.2 Sterilization and germination of seeds

The fruits were carefully washed with tap water containing few drops of a Tween^®^ 20 (Sigma-Aldrich, St. Louis, MO, USA) at a concentration of 4mL L⁻¹ (0.4% v/v). The seeds were manually separated from the pulp. Surface-sterilization was carried out under aseptic conditions. To surface-sterilize the seeds, they were soaked for 7min in a 2% available NaOCl solution prepared by diluting a 4% commercial sodium hypochlorite solution (1:1,v/v) with sterile distilled water, followed by three rinses with sterile distilled water, each lasting 10 min, to remove residual disinfectant. A total of 360 seeds were used, with each seed cultured individually in a glass test tube (2.5cm in diameter × 15cm in height) containing 15mL of culture medium. Each test tube was considered an experimental replicate, and each treatment was replicated 40 times. The test tubes were closed with their standard closures. Sterilized seeds were initially cultured on basal Murashige and Skoog (MS) medium without plant growth regulators, solidified with 6×10^3^mg L⁻¹ agar and incubated at 25 ± 1 °C in darkness (Koufan et al., 2020).

This helped in identifying and excluding contaminated or dead seeds from further processes of germination. Contamination was defined as the visible presence of microbial growth, including fungal or bacterial growth, on the culture medium or seed, whereas necrosis was defined as visible browning and tissue death of the seed or developing seedling. Germination was recorded upon radicle emergence. After germination they are transferred into a Murashige and Skoog culture medium at half strength MS/2 (applies to all salts and vitamins) (Murashige and Skoog, 1962) supplemented with 2mgL⁻¹ of gibberellic acid (GA_3_), The seedlings were then cultured in a growth chamber for 8 weeks.

The basal half-strength Murashige and Skoog MS/2 medium consisted of MS macro-elements, MS micro-elements, and MS vitamins, and was supplemented with 3% (w/v) sucrose. All culture media were solidified with 6×10^3^mg L⁻¹ agar. The pH of the medium was adjusted to 5.7 prior to autoclaving at 121 °C for 20 min. All cultures were maintained under a 16h photoperiod (light intensity of 40 μmol m⁻² s⁻¹) at 25 ± 1 °C and were transferred to fresh medium at 4-week intervals.

### 2.3 Scion preparation

The scions used were obtained from the apical shoots (micro-cuttings) of *in vitro*-grown plantlets of three genotypes (G41, G27, and G27/41), the latter being a product of a previous *in vitro* micrografting. The donor plantlets had been maintained in the laboratory under controlled environmental conditions for four years (donor-line maintenance) through successive subcultures on suitable culture media. The leafy shoots were excised to a length of 5–10mm, had a stem diameter of 2–3mm, and contained two to three nodes (**Table 1**).

### 2.4 Micrografting process

The rootstocks developed during germination were placed under a laminar flow hood for the simple apical cleft micrografting. The rootstocks were initially germinated in glass test tubes containing basal MS medium without plant growth regulators. Germination occurred between the first and second weeks of culture. After germination, the seedlings were transferred to half-strength MS (MS/2) medium supplemented with 2mg L⁻¹ GA_3_ and cultured for an additional 6 weeks, resulting in a total culture period of 8 weeks, after which the seedlings reached an appropriate developmental stage for micrografting. During the micrografting process, We started by removing the cotyledons, and a simple apical cleft, 5mm deep, was then created at the top of the rootstock. The scions are derived from *in vitro*-grown plantlets maintained in a growth chamber (previously cultivated micro-cuttings). Also, under the laminar flow hood apical micro-cuttings were collected and prepared as scions measuring 5–10mm in length, bearing 2–3 nodes and having the same stem diameter as the rootstocks (2–3mm), ensuring proper cambial alignment. The lower end of the scions was then cut into a V-shape (double bevel cut) at the base and inserted into the incision made at the top of the rootstock. After the scion was inserted into the rootstock, a small amount of gelled culture medium was applied around the graft union to maintain local humidity and stabilize the graft. Once the rootstock and scion were assembled, the grafted plants are then transferred into glass test tubes containing MS/2 medium supplemented with 0.1mg L⁻¹ GA_3_, 0.5mg L⁻¹ GA_3_, or 0.5mgL⁻¹ GA_3_+0.5mg L⁻¹ BAP.

### 2.5 Statistical analysis

Five response variables were analyzed: (1) seed germination, (2) shoot regeneration and shoot-number distribution, (3) graft success/necrosis, (4) shoot length, and (5) the overall multivariate response structure. Seed germination was assessed with n = 40 replicate seeds per run, repeated across several independent runs, and outcome categories (germination/contamination/necrosis) were compared with a one-way ANOVA and Tukey’s HSD *post-hoc* test in GraphPad Prism 10.6.1, consistent with the error bars and significance brackets. For shoot regeneration, shoot-number categories, and shoot length, the experimental design was a fully crossed factorial with three scion lines (GENOTYPE: G41, G27, G27/41) and three phytohormonal media (MILIEU: 0.1 mg L⁻¹ GA3, 0.5 mg L⁻¹ GA3, 0.5 mg L⁻¹ GA3 + 0.5 mg L⁻¹ BAP), giving scion line × medium combinations with n = 40 explants each (N = 360), one explant per culture vessel, vessels randomly allocated to each medium. To achieve the required number of germinated rootstocks and successful grafts, the experiment was spread across 9 independent experimental runs using a total of 360 germinated G54 donor plants as rootstocks. Graft (micrografting) success and necrosis were recorded and analyzed strictly across the three scion lines, pooling data independently of the phytohormonal media (N = 120, n = 40 per scion line), and compared using a one-way ANOVA with Tukey’s HSD *post-hoc* test in GraphPad Prism 10, consistent with the error bars and significance brackets.

For the response variables analyzed in IBM SPSS Statistics v27, the relevant factors were tested jointly rather than as *post-hoc* pairwise comparisons. Binary outcomes, including overall shoot regeneration along with the specific shoot-number classes (New shoots %, Single shoot %, Two shoots %, and > Two shoots %), were analyzed with a binomial generalized linear model (logit link) using Likelihood Ratio Omnibus tests to evaluate the main effects, and results are expressed as odds ratios with 95% confidence intervals, because this model represents the sampling variance of proportion data directly. Shoot length (cm) was analyzed with a two-way ANOVA (Type III sums of squares); homogeneity of variance was assessed with Levene’s test and addressed with a robust Welch sensitivity analysis, and significant main effects were followed by Bonferroni-adjusted pairwise comparisons of estimated marginal means. Effect sizes (partial η² for ANOVA; odds ratios for GLM models) and 95% confidence intervals are reported alongside all p-values.

A principal component analysis (PCA) was performed on 5 standardized response variables (New shoots, One shoot, Two shoots, More than two, and Length), based on the 120 fully monitored grafted plants as the unit of observation, to summaries the overall pattern of variation among scion lines (PC1 = 31.6%, PC2 = 27.2% of total variance). All analyses were performed in IBM SPSS Statistics v27 (α = 0.05), except seed germination and graft success/necrosis, which were analyzed in GraphPad Prism 10.6.1, and the principal component analysis, which was performed in OriginPro 2026.

### 2.6 Histological analysis

After two months of the micrografting process, graft-union samples were collected from the best-performing G41/G54 combination for histological studies. The sample was taken from the graft union region, and 0.5 cm of tissue was collected above and below the grafting point to ensure that the graft interface was among the materials under study. The samples were then fixed in FAA (formaldehyde, acetic acid, and ethanol) for 48 h, dehydrated through a graded series of ethanol (10%, 30%, 50%, 70%, 90%, and 95%) for 60 min at each concentration, and subsequently immersed in 100% ethanol overnight. The samples were embedded in paraffin and sectioned transversely into 7-μm-thick sections using a rotary microtome (Leica RM2245, Germany). The sections were double-stained with Safranin O and Fast Green and examined under a light microscope (Leica DMLS, Germany). Representative micrographs were obtained to qualitatively characterize the anatomical organization of the graft union and assess vascular reconnection and vascular bridge formation between the scion and rootstock. As the histological analysis was conducted only for the best-performing G41/G54 combination and at a single time point, the observations were considered qualitative and were not used for direct quantitative comparisons of compatibility among the different graft combinations.

## 3 Results

### 3.1 Rootstock preparation

**Figure 1:**
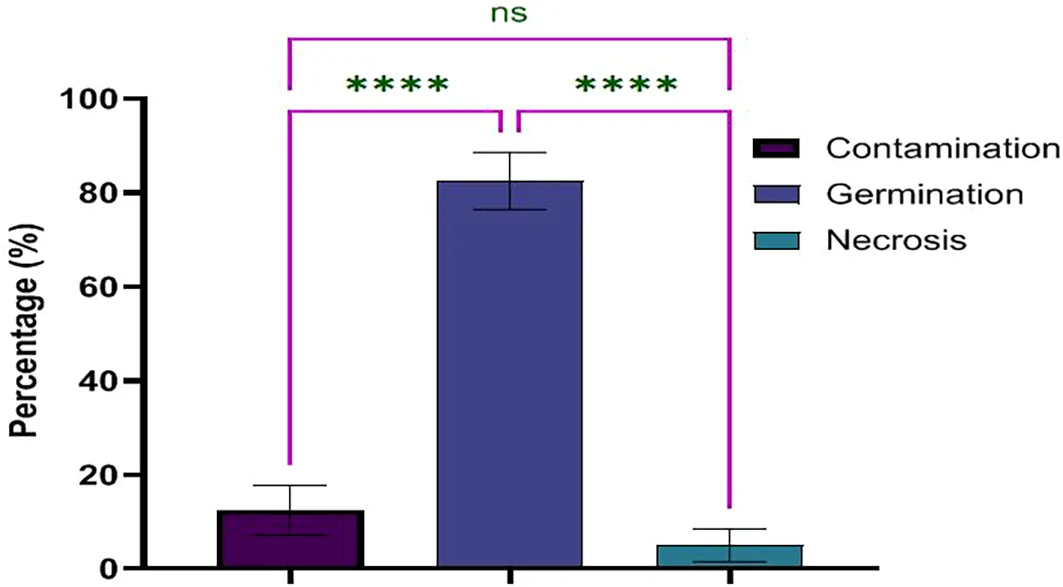
Germination success rate of argan tree seeds. **** p < 0.0001.

An important step in rootstock production is the *in vitro* germination of the argan tree. The seeds were surface sterilized first before being put on basal Murashige and Skoog (MS) medium without plant hormones and incubated in dark conditions at a temperature of 25 ± 1 °C. Germination was recorded upon radicle emergence and occurred between the first and second weeks of culture. A total of 360 seeds were used in nine independent experiments, with 40 seeds per replicate. At the end of the germination stage, 295 seeds germinated (82%), 43 seeds were contaminated (12%), and 22 seeds showed necrosis (6%). A one-way ANOVA with Tukey’s HSD *post-hoc* test confirmed that the germination rate was significantly higher than both the contamination and necrosis rates (**Figure 1**), while contamination and necrosis rates did not differ significantly from each other. Then, the resulting germinated seedlings were put on half-strength MS (MS/2) medium with addition of 2mg L⁻¹ GA_3_ and provided with conditions for germination from sowing until it was ready for micrografting. These conditions supported seed germination and production of seedlings suitable for the subsequent micrografting experiment.

**Figure 2:**
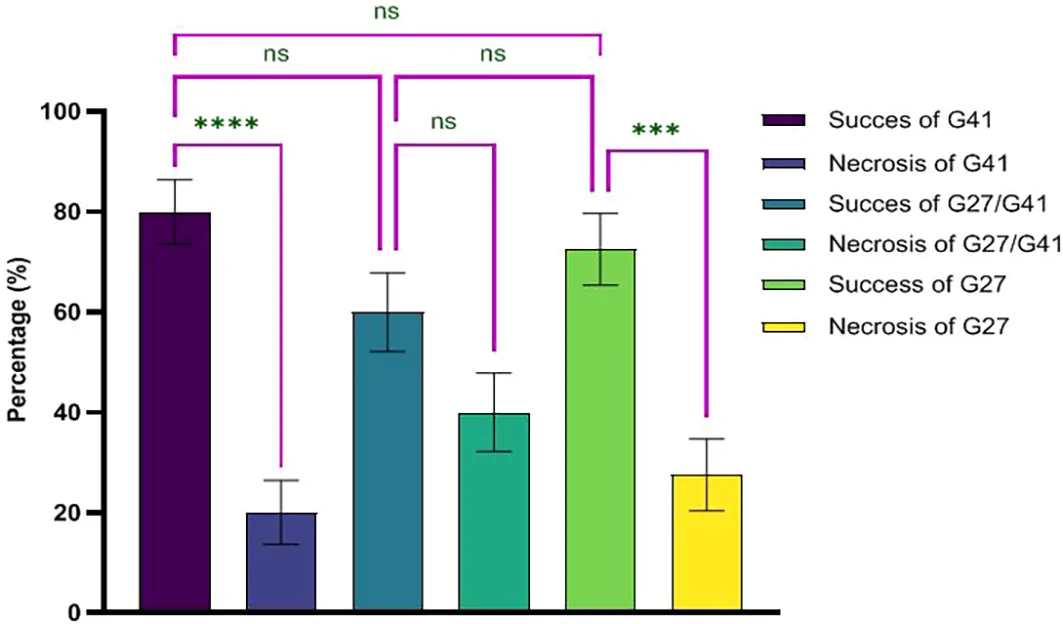
Comparative analysis of graft success and necrosis rates of three scion genotypes (G41, G27, and G27/41) grafted onto rootstock G54. *** p < 0.001; **** p < 0.0001.

### 3.2 Genotypic variation in *in vitro* shoot regeneration

The results reveal significant variation in regeneration parameters depending on genotype and culture medium (**Table 2**). Overall, genotype G41 shows the highest performance, with a new shoot formation rate reaching 100 ± 0.00% under 0.1 mg L⁻¹ GA_3_, followed by 95 ± 22.07% under 0.5 mg L⁻¹ GA_3_. In contrast, genotype G27 exhibits the lowest values, with a maximum of 60 ± 49.61%. Genotype G27/41 displays intermediate responses, ranging from 60 ± 49.61% to 75 ± 43.48%, depending on the treatment. Regarding shoot type distribution, the binomial generalized linear model (GLM) demonstrated highly significant main effects for both genotype (LR ***χ²* **=54.342, *df* = 6, *p* <.001) and milieu (LR ***χ²* **=79.114, *df* = 6, *p* <.001) within a robust additive framework (Model ***χ²* **=133.393, *df* = 12, *p* <.001, **Table 3**). Contrast profiling confirmed that G41 possessed a substantially higher likelihood of regeneration than the G27/41 reference line (Odds Ratio [OR] = 3.78, 95% CI [1.29, 11.06], *p* = .015). Under these models, the 0.1 mg L⁻¹ GA_3_ treatment mainly promotes single shoot formation, reaching 70 ± 46.41% in G41 and 62 ± 49.02% in G27/41. In contrast, the combination of 0.5 mg L⁻¹ GA_3_ and 0.5 mg L⁻¹ BAP enhances multiple shoot formation, particularly structures bearing more than two shoots, with values reaching up to 20 ± 40.51% in G41 and 19 ± 40.51% in G27. For the two-shoot category, values range from 8 ± 27.75% to 35 ± 48.39%, with the highest levels recorded in G41 under 0.5 mg L⁻¹ GA_3_.

**Table 2:** Descriptive statistics of shoot regeneration parameters and mean shoot length of *Argania spinosa* L. across different treatment combinations (Genotype × Growth regulator).

Genotype	Treatments (mg L⁻¹)	New shoots (%)	Single shoot (%)	Two shoots (%)	> Two shoots (%)	Mean length (cm)
G41	0.1 GA_3_	100 ± 0.00	70 ± 46.41	30 ± 46.41	0 ± 0.00	2.8 ± 0.64
	0.5 GA_3_	95 ± 22.07	60 ± 49.61	35 ± 48.39	3 ± 15.81	2.9 ± 0.07
	0.5 GA_3_ + 0.5 BAP	86 ± 36.16	16 ± 37.42	32 ± 47.43	20 ± 40.51	2.6 ± 0.65
G27/41	0.1 GA_3_	75 ± 43.48	62 ± 49.02	10 ± 26.67	0 ± 0.00	2.5 ± 0.70
	0.5 GA_3_	69 ± 46.71	58 ± 49.50	16 ± 37.42	1 ± 12.85	2.8 ± 0.64
	0.5 GA_3_ + 0.5 BAP	60 ± 49.61	20 ± 40.16	25 ± 43.85	18 ± 38.48	2.2 ± 0.94
G27	0.1 GA_3_	55 ± 50.38	60 ± 49.61	8 ± 27.75	0 ± 0.00	2 ± 1.19
	0.5 GA_3_	59 ± 49.50	45 ± 49.95	10 ± 26.67	0 ± 0.00	2.2 ± 0.94
	0.5 GA_3_ + 0.5 BAP	60 ± 49.61	25 ± 43.85	16 ± 37.42	19 ± 40.51	1.8 ± 1.14

Values are expressed as mean ± standard deviation (N = 360 explants total, n = 40 per scion line × medium combination).

**Table 3:** Binomial GLM likelihood ratio tests and contrast analyses for shoot parameters in *Argania spinosa*.

Parameter/contrast	Statistic	*df*/95% CI	*p*-value	Odds ratio (OR)
Omnibus Additive Model	*χ²* = 133.393	12	<.001	*Nagelkerke R^2^=0.338*
Genotype (Main Effect)	LR *χ²* = 54.342	6	<.001	—
Milieu (Main Effect)	LR *χ²* = 79.114	6	<.001	—
*G41 vs. G27/41 (Ref.)*	Contrast	[1.29, 11.06]	0.015	3.78
*G27 vs. G27/41 (Ref.)*	Contrast	[0.41, 2.45]	1.000	1.000
*0.1* mg L⁻¹ GA_3_ vs. Mixture (Ref.)*	Contrast	[0.34, 1.98]	0.651	0.820
*0.5* mg L⁻¹ GA_3_ vs. Mixture (Ref.)*	Contrast	[0.41, 2.45]	1.000	1.000

*N* = 360 observations (*n* = 40 per treatment combination). LR *χ²*, Likelihood Ratio Chi-Square test; OR, Odds Ratio; CI, Confidence Interval; *df*, degrees of freedom. Ref. indicates the designated reference category used for calculating the contrast constraints. Bold *p*-values indicate statistical significance at (α = 0.05). *Mixture reference category corresponds to 0.5 mg L⁻¹ GA_3_ + 0.5 mg L⁻¹ BAP.

Mean shoot length, analyzed with a two-way ANOVA (Type III sums of squares), shows limited variation among genotypes and treatments, ranging from 1.8 ± 1.14 cm to 2.9 ± 0.07 cm, with the maximum value observed in G41 under 0.5 mg L⁻¹ GA_3_. The global corrected model was highly significant (*F*_(8, 351)_ = 19.787, *p* < 0.001, **Table 4A**), heavily driven by the main effects of genotype (*F* = 67.236, *p* < 0.001, Partial *Ƞ^2^* = 0.277) and milieu (*F* = 9.212, *p* < 0.001, Partial *Ƞ^2^* = 0.050), while their interaction remained non-significant (*F* = 1.349, *p* = 0.251). Bonferroni *post-hoc* comparisons (**Table 4B**) confirmed that G41 produced significantly longer shoots than G27 (mean difference = 0.892 cm, *p* < 0.001) and G27/41 (mean difference = 1.424 cm, *p* < 0.001). Generally, these results demonstrate quantitative differences between different genotypes and hormonal treatments regarding all parameters.

**Table 4a:** Two-way ANOVA summary for mean shoot length in *Argania spinosa*.

Source	SS	df	MS	*F*	*p*-value	Partial η²
Corrected Model	146.290	8	18.286	19.787	< 0.001	0.311
Genotype (Scion Line)	124.275	2	62.138	67.236	< 0.001	0.277
Milieu (Hormonal Medium)	17.027	2	8.513	9.212	< 0.001	0.050
Genotype × Milieu	4.988	4	1.247	1.349	0.251	0.015
Error	324.381	351	0.924			

*N* = 360 observations. SS, Type III Sum of Squares; MS, Mean Square; F, Fisher’s *F*-statistic; Partial η², Partial eta-squared effect size. Bold *p*-values indicate statistical significance at α = 0.05.

**Table 4b:** Bonferroni *post-hoc* pairwise comparisons for mean shoot length in *Argania spinosa*.

Factor	Comparison	Mean difference (cm)	95% confidence interval	*p*-value
Genotype	*G41 vs. G27*	0.892	[0.593, 1.190]	< 0.001
	*G41 vs. G27/41*	1.424	[1.126, 1.723]	< 0.001
	*G27 vs. G27/41*	0.532	[0.234, 0.831]	< 0.001
Milieu	*0.1* mg L⁻¹ *GA_3_ vs. 0.5 mg L⁻¹ GA_3_*	−0.091	[−0.389, 0.208]	1.000
	*0.1* mg L⁻¹ GA_3_ vs. Mixture*	0.409	[0.111, 0.708]	0.003
	*0.5* mg L⁻¹ GA_3_ vs. Mixture*	0.500	[0.201, 0.799]	< 0.001

Pairwise comparisons are based on estimated marginal means followed by a Bonferroni adjustment for multiple testing. Bold *p*-values indicate statistical significance at α = 0.05. *Mixture reference category corresponds to 0.5 mg L⁻¹ GA_3_ + 0.5 mg L⁻¹ BAP.

**Figure 3:**
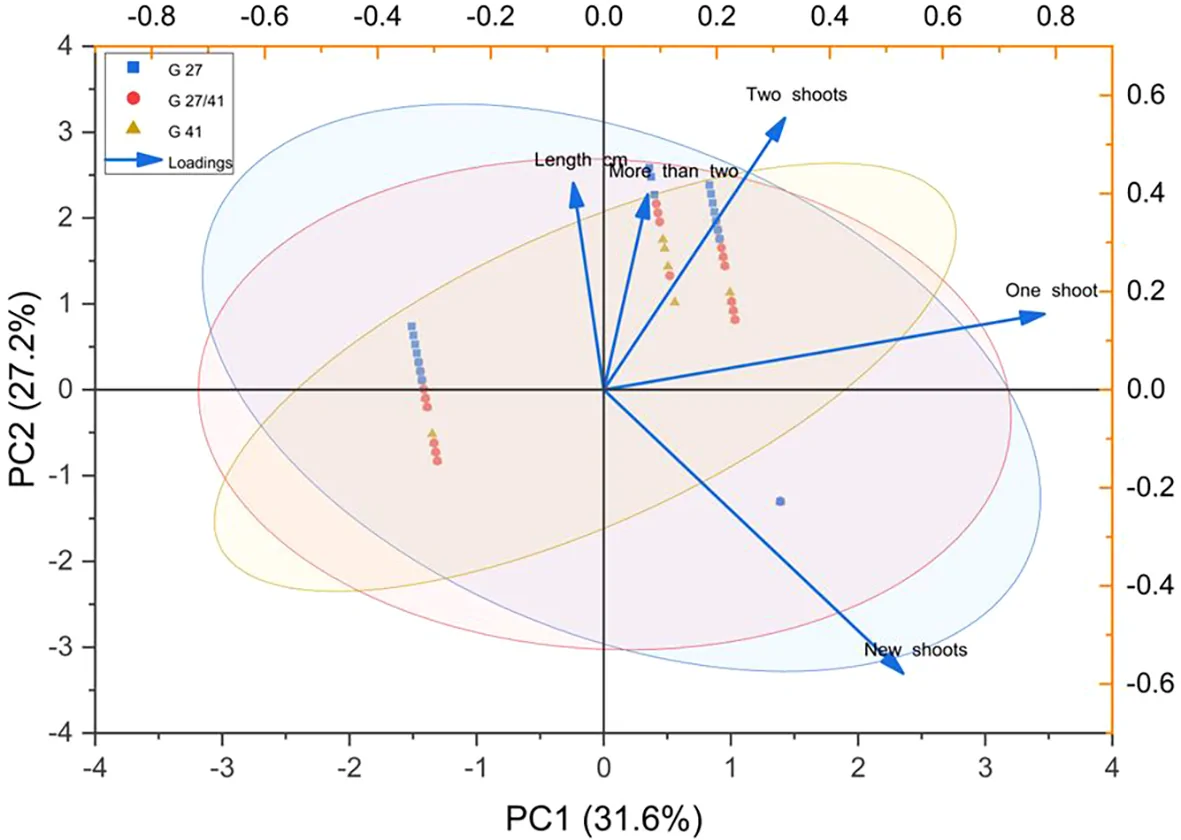
Principal component analysis (PCA) of the *in vitro* morphogenetic response of the investigated genotypes.

The multivariate spatial projection of growth attributes via PCA (**Figure 3**) successfully captures 58.8% of the total dataset variance within its first two dimensions. Principal Component 1 (PC1) accounts for 31.6% of the total variance (Eigenvalue = 1.58) and serves as a general “overall morphogenetic response” axis: all five variables show positive loadings on this component, with “One shoot” showing the strongest, most nearly horizontal loading and therefore contributing most strongly to this component; explants/combinations positioned further to the right of the biplot display generally stronger responses across the measured variables. Principal Component 2 (PC2) explains an additional 27.2% of the cumulative variance (Eigenvalue = 1.36) and acts as a “progression/maturity” axis, showing contrasting loading patterns where shoot length and the multi-shoot classes (Two shoots, More than two) load positively (upward), whereas the New-shoots indicator loads negatively (downward); combinations located higher on PC2 tend to exhibit longer and more numerous shoots, while those located lower on PC2 remain more associated with newly emerging shoots. The 95% confidence ellipses for the three scion lines in **Figure 3** overlap substantially; separation between lines is therefore observed mainly at the centroid level along PC1 rather than as fully distinct, non-overlapping clusters. Accordingly, G41 shows a stronger association with morphogenetic traits related to shoot elongation and branching potential, suggesting greater association with advanced shoot development. G27 is positioned closer to the New-shoot variable, indicating a tendency toward shoot initiation rather than advanced structural development. G27/41 occupies an intermediate position, reflecting a moderate response across the measured parameters without the stronger elongation and branching tendency observed for G41. Overall, PC2 reflects a gradient between early shoot emergence and more advanced shoot development, rather than a complete separation of genotypes into distinct phenotypic groups.

### 3.3 Micrografting compatibility

The grafting response varied substantially and was highly dependent on the scion genotype used, as evaluated via a one-way ANOVA with Tukey’s *post-hoc* test. At eight weeks after micrografting, graft success was defined as survival with sustained scion growth and an intact graft union, whereas necrosis was defined as browning and tissue collapse at the graft union. When grafting scion G41 onto rootstock G54, the highest performance was registered, with a success rate of 80% and the lowest necrosis rate of 20% (**Figure 2**). These results indicate the highest observed grafting success among the tested scion lines under the evaluated conditions. However, grafting scion G27/41 onto rootstock G54 was less successful, as the success rate dropped to 60% and the necrosis rate was recorded at 40%. Medium compatibility was observed for scion G27 grafted onto rootstock G54, resulting in a success rate of 73% and a necrosis rate of 27%, thus showing an intermediate level of compatibility compared to the other scion genotypes tested. These results suggest that micrografting success varied among the scion genotypes tested, with statistically significant differences observed among combinations (p < 0.001). Among all combinations investigated, G41/G54 showed the highest observed success rate under the evaluated conditions.

### 3.4 Histological analysis

**Figure 4:**
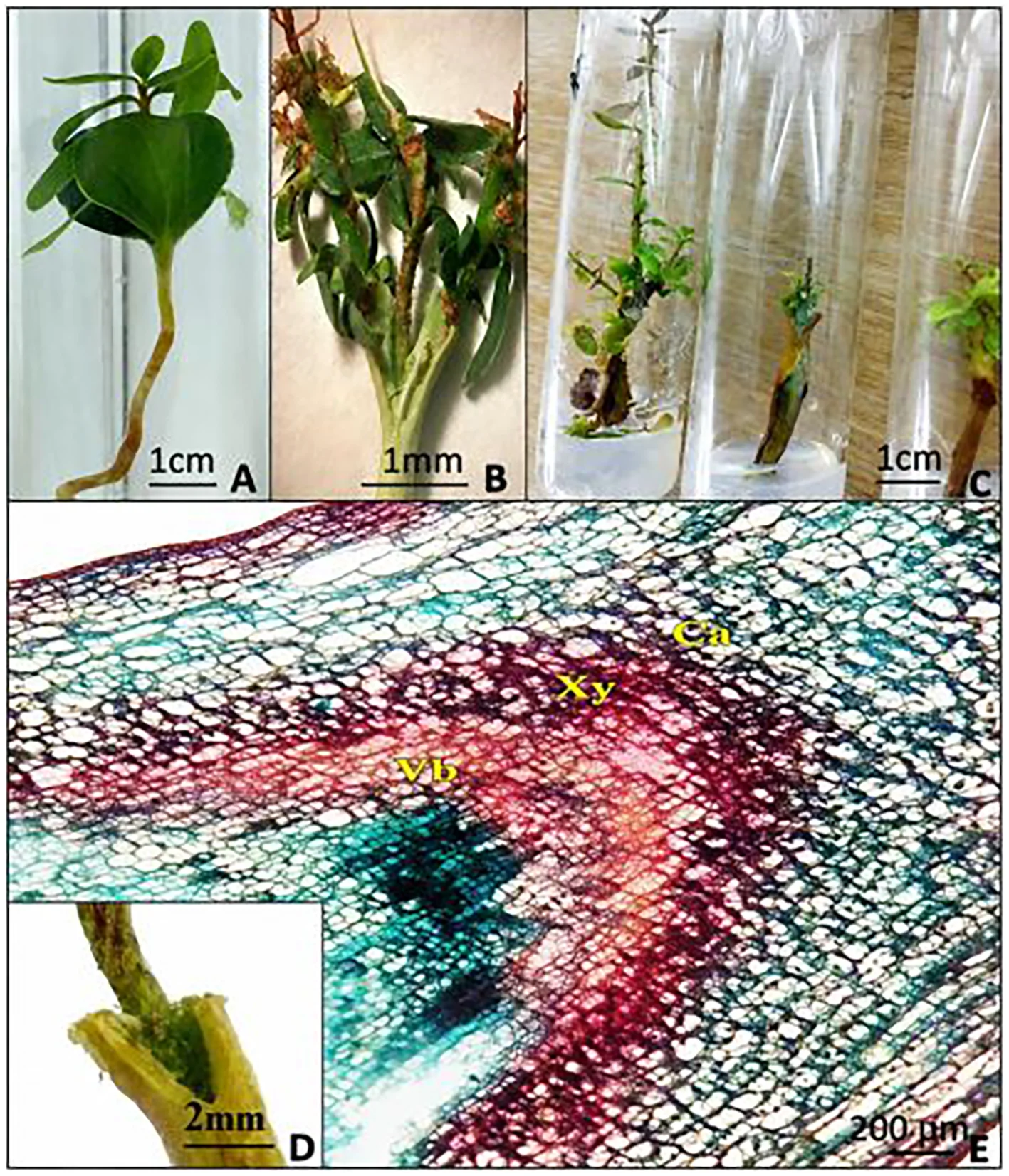
**(A)** *In vitro* germination of the rootstock, showing well-developed roots and hypocotyl prior to grafting. **(B)** Preparation of the scion and establishment of contact between the grafting partners under sterile conditions. **(C)** Sequential stages of graft development and shoot elongation in culture tubes, illustrating the development of the aerial part. **(D)** Macroscopic view of the graft union, demonstrating successful mechanical establishment and the formation of the initial callus at the interface between the scion and the rootstock. **(E)** Transverse histological section of the graft union stained with Safranin O and Fast Green. The micrograph reveals successful vascular reconnection, with lignified xylem vessels (Xy) stained red and the active cambial zone (Ca) stained green. The establishment of a vascular bridge (Vb) confirms high tissue compatibility and successful integration between the two genotypes (10× objective). Xy, xylem; Ca, cambium; Vb, vascular bridge.

The microscopic study of the graft junction between scion G41 and rootstock G54 (G41/G54 combination) showed very well-formed and uniform tissue integration, which is indicative of a successful compatible grafting (**Figure 4**). Histologically, the samples were stained with Safranin O and Fast Green, which provided a clear differentiation of the cellular structures: the lignified tissues, including the mature xylem vessels and tracheary elements, were sharply defined by the intense red of the Safranin, while the non-lignified components such as the phloem, cambium, and cortical parenchyma exhibited a vibrant green contrast from the Fast Green. This double staining revealed callus continuity across the graft interface, with cambial-like cell organization and lignified xylem elements spanning the union. These anatomical features are consistent with graft-union formation; however, functional water or solute transport was not assessed. Additionally, the interface lacked any fragmented necrosis layers or substantial interruptions of parenchyma tissue, both of which hamper hydraulic conductivity. Moreover, the presence of neatly arranged red secondary xylem without any abnormality along the interface point is consistent with anatomical integration of the G41/G54 combination.

## 4 Discussion

The argan tree (*Argania spinosa* (L.) Skeels) is an ecological and socio-economic entity within the southwestern region of Morocco. Due to its endangered status as a species and designation as a UNESCO Biosphere Reserve since 1998, the argan tree is increasingly threatened by deforestation, overgrazing, and climate change (Mazri et al., 2022; Tesse et al., 2024). The overarching goal of this research was to establish an argan tree regeneration system based on micrografting, a hybrid method combining the benefits of traditional grafting and *in vitro* tissue culture methods. Traditional propagation methods (sexual reproduction using seeds, propagation using cuttings, and conventional grafting) have not proven successful to propagate this candidate species (Nouaim et al., 2002; Bousselmame et al., 2001) and previous studies utilizing micropropagation through the culture of axillary buds or organogenesis also did not demonstrate sufficient success, especially at the rooting and acclimatization stages (Koufan et al., 2018; El-Qadmi et al., 2023). Micrografting is proposed as a highly viable method of conserving and producing argan trees by combining the vigorous growth and rooting ability of seedling rootstocks with the genetic characteristics of a selected scion.

In our experiments, the argan rootstocks produced from seeds germinated fully *in vitro* on basal MS medium without plant growth regulators achieved an 82% germination rate; this is similar to what has been reported by (Mezghenni et al., 2014) using 1/2 strength Murashige and Skoog medium (MS/2) supplemented with sucrose. These findings indicate that basal MS medium is suitable for producing *in vitro* rootstocks. The use of *in vitro*-germinated seeds as a starting point for the vegetative propagation of argan was also demonstrated by (Justamante et al., 2017), providing further evidence supporting the potential of this approach for argan propagation.

Surface sterilization of the seeds is an important step for *in vitro* culture that establishes the amount of uncontaminated material that will be available for future grafting. There are several established protocols for argan. (Mezghenni et al., 2014) provided a multi-step protocol that uses Tween soaking, the commercial fungicide Benlate at 1 g L^-1^, and mercuric chloride at 1 g L^-1^, with extensive rinsing with sterile distilled water. (Koufan et al., 2018) used only sodium hypochlorite (NaOCl) as the primary disinfectant. One would expect the variability seen between different studies to be related to the respective origin of seeds and the extent of endogenous microbial contamination since these parameters can differ depending on collection site, harvest time, and seed storage conditions (Berka et al., 2021). In the present study, our laboratory has used a diluted sodium hypochlorite and established a high proportion of aseptic, viable seeds similar to the method validated by (Koufan et al., 2018). Thus, we strongly recommend laboratories adhere to standard sterilization protocols as a first step in producing reproducible root stocks.

Seedlings were used as rootstocks for micrografting after approximately 8 weeks of culture, including 1–2 weeks for germination followed by 6 weeks of growth on half-strength MS (MS/2) medium supplemented with 2 mg L⁻¹ GA_3_, when they had reached an appropriate developmental stage for grafting. The rootstock genotype G54 was used for all micrografting experiments, while G41, G27, and G27/41 were used as scion genotypes. A simple apical cleft micrografting technique was employed, in which the cotyledons were removed and the rootstock was cut just below the cotyledons. A vertical cleft approximately 0.5 cm deep was then made at the apex of the rootstock, into which the V-shaped (double-bevel) basal end of the scion was inserted. This wedge method was previously validated with pistachio (*Pistacia vera* L. cv. Mateur) when (Chatibi, 1998) reported grafting success rates of 67% with some experimental conditions and 76% with others. Grafting success rates were 80% for G41/G54, 73% for G27/G54, and 60% for G27/41/G54, indicating differences in grafting performance among the scion genotypes when grafted onto the same G54 rootstock. Confirmation by (Carloni et al., 2025) demonstrates that scions with similar frequencies of sprouting were possible with *Neltuma alba* using micrografting and *in vitro* seedling rootstocks confirming that this technique can be replicated between species that are usually difficult to propagate by conventional methods. The use of nodal segments rather than shoot tips was influenced by the availability of this explant type and the potential of this explant type to produce axillary buds under *in vitro* culture conditions (Koufan et al., 2018; Ashrafzadeh, 2020).

Histological examination provided anatomical evidence of active vascular-tissue development bridging scion and rootstock. The anatomical features observed at two months correspond to structures associated in the literature with callus formation, cambial differentiation and vascular reconnection. The co-occurrence of callus proliferation at the graft site, differentiated procambial strands, and vascular tissues spanning the graft interface is consistent with the five developmental stages of graft-union formation described in the literature: necrotic-layer formation, callus-cell proliferation, callus-bridge formation, vascular-cambium formation, and xylem/phloem reconnection (Pina and Errea, 2005). In passionfruit, vascular connections form approximately 20 days after micrografting, after which scion growth accelerates markedly (Ribeiro et al., 2015). Our histological observations are consistent with those of Koufan et al. (2020), who documented cellular proliferation and vascular reconstruction at the argan graft interface using serial anatomical sectioning, and align with similar vascular-bridge formation reported in other difficult-to-root woody species, including *Neltuma alba* (Carloni et al., 2025) and kiwifruit (Bao et al., 2020).

Tissue necrosis at the graft interface remains a leading cause of graft failure, in argan as in other species (Koufan et al., 2020). Potential mechanisms described in other graft systems include to the wound response that follows grafting, involving a rapid rise in reactive oxygen species and intracellular calcium that can trigger programmed cell death if unresolved (Zhao et al., 2021). Similarly, auxin redistribution at the graft junction has been proposed to influence callus formation and vascular reconnection if prolonged (Zhao et al., 2021; Tahtah et al., 2026a). However, reactive oxygen species, calcium signaling, and auxin distribution were not measured in the present study. Future work should examine whether adjustments to culture-medium renewal timing, antioxidant supplementation, or explant preconditioning can reduce necrosis frequency in argan micrografts.

This study has several limitations. Only one rootstock genotype (G54) was tested, and reciprocal or additional rootstock combinations are needed before broader genotype-dependent compatibility can be claimed. The graft-derived line G27/41 requires further genetic characterization. Histological analysis was performed on a single combination (G41/G54) and at a single time point (2 months post-grafting), which documents anatomical continuity but does not establish functional hydraulic conductivity. Regeneration data showed high variability among replicates, which was robustly handled by utilizing Likelihood Ratio Omnibus tests within an additive binomial generalized linear model framework (**Table 3**) to successfully circumvent the structural variance and quasi-complete separation limits often inherent to traditional Wald estimations for low-frequency proportion cells. Finally, no acclimatization, ex vitro survival, or long-term field performance data are yet available. Post-grafting hormonal treatments were not fully decoupled from genotype in the present design, which may confound genotype and medium effects. Future work should address these points before the protocol is applied scale.

Overall, the findings of this study support using micrografting as an alternative method of propagating the *Argania spinosa* (argan) plant. Microcutting is difficult to use because adult-derived explants have difficulty developing roots (Koufan et al., 2018; Mazri et al., 2022). However, micrografting does not have to deal with this root development stage since it utilizes fast-growing seedling rootstocks, which resolve a major obstacle in the *in vitro* culture of argan. Additionally, rootstocks can be produced from large quantities of seed harvested and the wedge grafting process can be incorporated into routine laboratory procedures, although it requires a level of technician skill. According to (El-Qadmi et al., 2023) and (Tesse et al., 2024), developing viable *in vitro* propagation systems for argan is an urgent conservation need given the continued decline in wild populations and increasing global demand for argan products. If validated through acclimatization, nursery-scale multiplication, and long-term field trials, micrografting protocols may ultimately contribute to clonal propagation and conservation programs by enabling the selection of superior scion genotypes, thereby supporting future ecological restoration of argan forests and the sustainable production of economically valuable argan-derived products.

## 5 Conclusion

This study provides preliminary evidence that *in vitro* germinated G54 seedlings can support micrografting of selected *Argania spinosa* scion genotypes, with G41 showing the highest graft success and favorable histological features of graft-union formation under the tested conditions. The histological observations demonstrate anatomical continuity but do not establish functional hydraulic transport. Further work involving additional rootstock genotypes, reciprocal grafting, comparative histology, acclimatization, and long-term survival assessment is required before the protocol can be recommended for large-scale clonal propagation or conservation use.

## Data Availability

The original contributions presented in the study are included in the article/supplementary material. Further inquiries can be directed to the corresponding author.
